# Structural Design and Performance Optimization of Proton Exchange Membranes for Water Electrolysis: A Review

**DOI:** 10.3390/membranes16020054

**Published:** 2026-01-31

**Authors:** Yi Chen, Hongyang Ma, Benjamin S. Hsiao

**Affiliations:** 1 State Key Laboratory of Organic-Inorganic Composites, Beijing University of Chemical Technology, Beijing 100029, China; 2 Department of Chemistry, Stony Brook University, Stony Brook, NY 11794-3400, USA; benjamin.hsiao@stonybrook.edu

**Keywords:** PEM, PEMWE, ionic conductivity, stability, composite, structure–performance relationship

## Abstract

The trade-off between the ionic conductivity and the stability of the proton exchange membrane (PEM) is a major concern in the development of PEM water electrolysis (PEMWE). This review focuses on the design and fabrication of homogeneous and composite PEMs for water electrolysis and establishes the structure–performance relationships between the membrane chemical/physical structures and their efficiency metrics—specifically, proton conductivity, hydrogen permeability, and chemical and mechanical stability. A special focus is placed on the fundamental connection between the microstructure and performance of membrane materials. At the molecular level, we systematically illustrate the design principles for main chains, side chains, and sulfonate groups, covering both fluorinated PEMs (encompassing perfluorinated and partially fluorinated membranes) and non-fluorinated PEMs (including aromatic polymers with heteroatom backbones and all-carbon backbones). At the macroscopic level, the review provides an in-depth exploration of two primary modification strategies: creating composites with organic polymers and with inorganic nanofillers. In summary, this review elucidates how these composite approaches leverage material synergies to improve the membrane’s mechanical integrity, proton conduction efficiency, and chemical resistance and offers a theoretical framework for the rational design of next-generation, high-performance PEMs to advance the commercialization of PEMWE technology.

## 1. Introduction

The accelerated growth of the global population and economic activities has led to a surge in energy needs. Over-reliance on fossil fuel consumption has triggered environmental and climate crises, such as the triad of greenhouse gas emissions, global warming, and extreme weather [1]. Green hydrogen serves as a crucial driver for the development of the renewable energy transition and helps reduce greenhouse gas emissions [2,3]. Hydrogen production by water electrolysis, as a clean and sustainable method for hydrogen generation, boasts significant advantages: its pollution-free and zero-emission nature can effectively address global warming. Meanwhile, it is highly compatible with diverse renewable energy technologies, such as photovoltaics and wind energy, and can properly solve the problems of intermittency and unpredictability in renewable energy power generation [4,5].

Water electrolysis generates hydrogen through the fundamental process of water decomposition, generating hydrogen gas at the cathode and oxygen gas at the anode. With no emissions, it is an environmentally friendly method of hydrogen production [6,7]. It can be mainly divided into four types of technologies: alkaline water electrolysis (AWE) [8], proton exchange membrane water electrolysis (PEMWE), solid oxide electrolysis (SOE) [9], and anion exchange membrane water electrolysis (AEMWE) [10]. These technologies differ from one another regarding electrolyte type, operating temperature, efficiency, and application maturity, with distinct focuses on technical priorities [11]. Among these, the PEMWE technology employs a PEM as the electrolyte and hydrogen ions (H^+^) as the charge carrier, typically operating at a mild temperature (20–80 °C). Compared with other technologies, PEMWE features high current density, high hydrogen purity, a compact system, fast response speed, and high-purity hydrogen production. It is therefore regarded as a premier technology in the green hydrogen sector [12,13,14].

A PEMWE is primarily composed of membrane electrode assemblies (MEAs), bipolar plates (BPs), and gas diffusion layers (GDLs) [15,16]. The MEA is the core region for electrolysis reactions, consisting of a PEM and anode/cathode catalyst layers. As a core component, the PEM plays a pivotal role in selectively allowing protons to pass through while preventing the cross-permeation of H_2_ and O_2_ gases. Its performance directly affects the proton conduction efficiency, gas tightness, mechanical stability, and long-term durability of the cell, making it a key component that connects the reactions at the anode and cathode [17,18,19]. Therefore, critical demands are raised for PEMs in water electrolysis: (1) It needs to have high proton conductivity, which should reach over 0.1 S/cm and remain stable under acidic aqueous and different temperature conditions [20]. (2) It must possess excellent gas barrier properties. Crossover of hydrogen and oxygen through the membrane can lead to forming hydroperoxides or free radicals, which in turn impairs the membrane’s chemical stability. Meanwhile, excellent gas barrier properties help enhance hydrogen production efficiency, prevent a decline in hydrogen purity, and avoid potential safety hazards [21,22]. (3) It must have strong chemical and electrochemical stability, enabling it to withstand strong acidic environments, high oxidation potential conditions, and strong oxidizing species. During long-term electrolysis, it should not undergo chemical degradation or participate in electrode reactions [23]. (4) It needs to have good dimensional stability and a low swelling rate to prevent structural damage caused by water absorption and swelling [24]. (5) It must have good thermal stability, maintaining structural stability at 60–80 °C or even higher temperatures.

**Figure 1:**
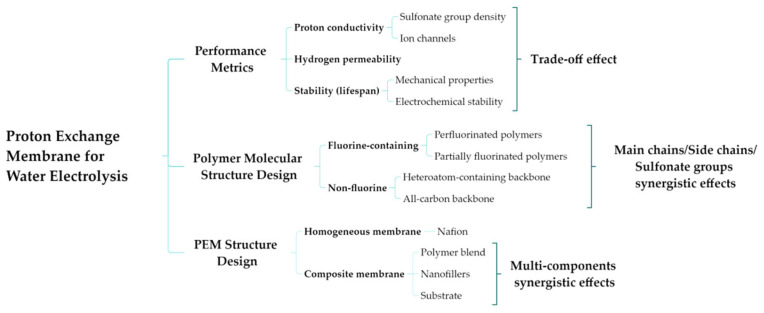
A mind map of proton exchange membranes for water electrolysis.

The performance of the PEM is the core guarantee for the efficient and stable operation of PEMWE. These properties are interrelated while also having certain trade-offs (e.g., high ionic conductivity may reduce dimensional stability) [17]. At present, the focus of research lies in achieving a balance among various properties by designing different material structures—including main-chain/side-chain structures and composite structures—to reduce costs, extend service life, adapt to a wider range of operating conditions (e.g., high temperatures), and minimize hydrogen permeation [25], as shown in Figure 1.

In hydrogen production via water electrolysis, the performance of PEMs is closely linked to their microstructure, with the core reflected in two key aspects: main-chain/side-chain design and homogeneous/composite membrane structure. On the one hand, the chemical stability of the main chain and the distribution of its side chains directly determine the membrane’s mechanical strength, proton conductivity, and chemical stability. On the other hand, introducing inorganic nanoparticles, such as silica and graphene oxide, or polymer support layers (e.g., PTFE) into the polymer matrix to form a composite structure can effectively make up for the shortcomings of homogeneous membranes in mechanical strength, thermal stability, and durability, thereby achieving synergistic optimization of performance. Therefore, the current focus of research is centered on two areas: first, designing novel polymer backbones that simultaneously possess high proton conductivity and excellent chemical stability, and second, developing more sophisticated composite membrane structures. Breakthroughs in these two directions can enable PEMs to operate efficiently and durably under harsh working conditions, providing crucial support for the large-scale application of clean hydrogen energy.

It should be noted that, different from existing reviews, which were mainly focused on proton exchange membranes (PEMs) and their fuel cell (PEMFC) applications, this review specifically addresses PEMs for water electrolysis (PEMWE) for hydrogen production, highlighting a distinct set of challenges and perspectives. Furthermore, it has been organized based on a mind-map framework that establishes structure–performance relationships, with the aim of breaking through the trade-off effects.

## 2. Performance Metrics

Proton conduction in PEMs is a complex process involving the synergistic action of multiple mechanisms. Within the nanoscale hydrophilic channels, protons primarily exist in the form of dynamically changing hydrated proton clusters, such as H_3_O^+^, H_5_O_2_^+^, and H_9_O_4_^+^, and are transferred via an efficient “Grotthuss mechanism” in a relay-like fashion. This mechanism is triggered by configurational fluctuations of water molecules and serves as the dominant pathway under normal operating conditions. Concurrently, a less efficient “vehicular mechanism” also exists, where protons combine with water molecules to form hydrated protons that subsequently diffuse together. The conduction process begins with the dissociation of protons from sulfonic acid groups (-SO_3_H), which requires the participation of at least three water molecules. These water molecules can shield the electrostatic attraction between the hydrated protons and the sulfonate anions, thereby promoting migration. Under low degrees of hydration, Zundel ions (H_5_O_2_^+^) act as important “relay stations” for proton transfer [26,27,28].

Hydrogen permeability is a critical issue in PEMWEs that leads to efficiency degradation and safety risks [29]. Its mechanisms are complex and diverse, including the dissolution–diffusion of hydrogen in hydrophilic channels, convective transport with water flow, and the electro-osmotic drag mechanism. These processes are closely correlated with factors such as current density, temperature, pressure, water content, and polymer structure. In terms of polymer structure, shorter side chains lead to a higher hydrogen permeability, which is attributed to physical structural factors rather than intermolecular interactions. Although longer side chains increase free volume, they can suppress hydrogen crossover through more intense kinetic behavior [30]. The polymer’s equivalent weight (EW) also affects hydrogen crossover by altering the membrane’s microstructure. High-EW membranes reduce free volume and hinder diffusion through high crystallinity, whereas low-EW membranes reduce solubility by decreasing the hydrophobic PTFE domains and impede diffusion through tortuous aqueous channels [31]. Quantum mechanics (DFT) and molecular dynamics (MD) simulations further clarify the kinetic mechanism underlying the effect of side-chain length on hydrogen permeation in PFSA-based membranes (e.g., Nafion) [30]. Using PFSA models with different side-chain repeating units (y = 1, 2, 5, 8; side-chain length 4.66–5.98 Å) and a hydration level of λ = 3, simulations showed that membranes with shorter side chains exhibit significantly higher hydrogen penetration ratios—the hydrogen penetration depth of the longest side chain (y = 8) is 21.7% lower than that of the shortest one (y = 1). DFT calculations (PBE-GGA functional) revealed that the electrostatic and van der Waals interactions between hydrogen and side chains are extremely weak (binding energy: −0.005 to −0.02 eV), with negligible impact on permeation. Instead, physical factors dominate: longer side chains have a larger radius of gyration (1.5 times that of shorter ones) and higher diffusion coefficients of terminal atoms, leading to more intense “trembling behavior” that forms a physical barrier, hindering hydrogen penetration. This kinetic hindrance effect of longer side chains far outweighs the promotion of free volume increase, further confirming that side-chain length regulates hydrogen permeability through dynamic structural barriers. To mitigate hydrogen crossover, various strategies have been developed: nanostructural modification by adjusting chemical composition and fabrication processes; use of composite membranes with modified PTFE or hydrocarbon polymers; construction of barrier layers using polymers or 2D materials (e.g., graphene and boron nitride); design of layered structures, including gas recombination catalyst layers and 2D material interlayers; incorporation of inorganic fillers (e.g., functionalized graphene, MOFs); creation of cross-linked structures or organic filler composite membranes; and development of designable hydrocarbon polymers with excellent hydrogen barrier properties [32]. Future work needs to focus on gaining a deeper understanding of the crossover mechanisms, resolving interfacial compatibility issues, quantitatively investigating the impact of materials on hydrogen crossover, and exploring the phenomenon of anode oxygen supersaturation.

The degradation of PEMs primarily involves chemical and mechanical mechanisms. Chemical degradation is initiated by •OH and •OOH radicals from hydrolysis. These radicals erode the polymer’s carboxylic acid end groups, ether linkages (C-O-C) in the side chains, and C-S bonds. This triggers chain scission, functional group loss, and the release of HF and CO_2_ and can be accelerated by the formation of platinum bands due to dissolved platinum catalyst. Mechanical degradation stems from swelling and shrinkage induced by humidity cycling, cracks or tears caused by localized stresses (e.g., at edges or assembly defects), and creep-induced thinning under long-term compression [33,34,35,36]. Addressing the chemical degradation mechanisms of PEMs by developing novel membrane materials and structures with high stability is a frontier research direction in the field of materials design.

The proton conductivity, hydrogen crossover, and lifetime of a PEM exhibit the trade-off effects. The high density and hydration of sulfonic acid groups required for high proton conductivity, while providing high-speed channels for protons, also create convenient pathways for hydrogen molecule permeation and make the membrane’s chemical structure more susceptible to degradation by radical attacks. Meanwhile, modification strategies, such as creating composites or cross-linked structures, adopted to suppress hydrogen crossover or enhance chemical stability, may in turn introduce interfacial defects or reduce material toughness, thereby sacrificing mechanical lifetime. Therefore, addressing the trade-off effect is a major challenge for the innovation of next-generation PEMs for water electrolysis applications.

## 3. Polymer Molecular Structure Design

In PEMs, the main-chain structure is the fundamental determinant of core performance, directly governing the baseline levels of chemical stability, thermal stability, mechanical properties, and proton conductivity. Its chemical composition (such as chemical bond strength and element types) dictates the durability of the membranes under thermal and chemical stress; the rigidity of the molecular chain and the aggregated structure influence its mechanical properties. Additionally, the main chain provides a stable framework for the proton-conducting function of side chains, serving as a prerequisite for the membrane’s long-term reliable operation. Side chains, within the performance framework defined by the main chain, play a supplementary role: they can enhance the local flexibility of the membrane to optimize mechanical properties and balance mechanical strength and proton conductivity efficiency by adjusting their length and distribution. Based on the elemental composition of the main chain, PEMs are categorized into two major types: fluorine-containing and non-fluorine.

### 3.1. Fluorine-Containing Polymers

#### 3.1.1. Perfluorinated Fluorinated Polymers

**Figure 2:**
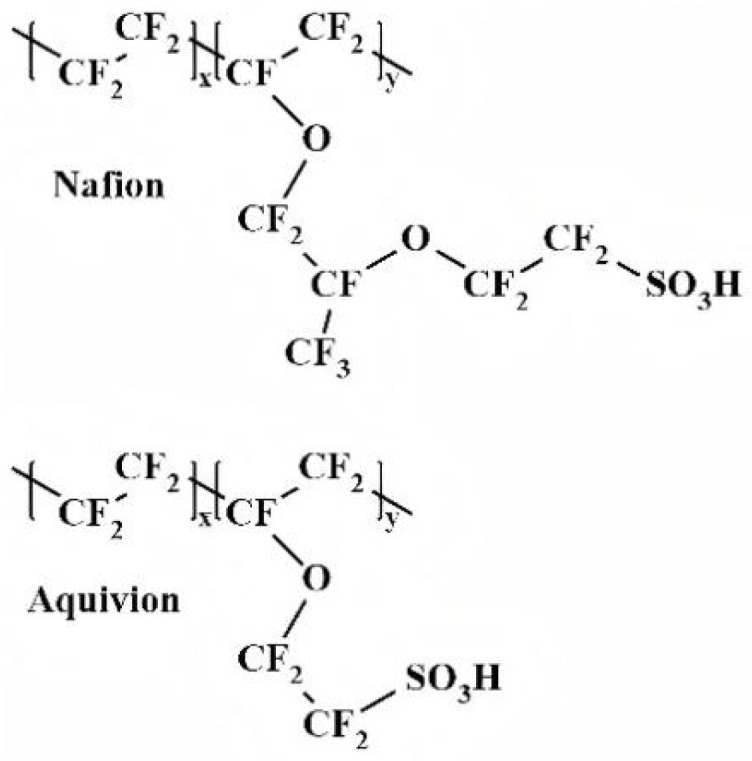
The structure of PFSA (Nafion® and Aquivion^®^).

Perfluorinated sulfonic acid (PFSA) resins are a class of polymers with high ionic conductivity and relatively high chemical/mechanical stability (with DuPont’s Nafion^®^ as a typical representative). The main chain of PFSA polymers is polytetrafluoroethylene (PTFE), while the side chains contain sulfonic acid groups (-SO_3_H). Phase separation of the hydrophilic and hydrophobic components yields interconnected proton-conducting channels, endowing the polymer with excellent chemical stability and proton conductivity [37]. Its performance is closely related to the side-chain length and equivalent weight (EW, the mass of dry polymer per mole of ionic groups). Nafion^®^ (by DuPont, Wilmington, DE, USA) is the most commonly used PFSA PEM. Different models, varying in thickness and equivalent weight, are suitable for different scenarios. Other products, such as Flemion^®^ [38] and Aquivion^®^ [39], have their respective performance focuses due to differences in side-chain length or functional group density (for example, the short-side-chain structure of Aquivion^®^ enhances stability) [40]. Balancing the side-chain length and equivalent weight of PFSA polymers to obtain PEMs with excellent comprehensive performance is currently a research focus. The structures of Nafion^®^ and Aquivion^®^ are illustrated in Figure 2.

The performance of PFSA is mainly determined by its side-chain length and EW [41]. According to the side-chain length, PFSA resins can be classified into long-side-chain (LSC) ones (e.g., Nafion^®^) and short-side-chain (SSC) ones (e.g., 3M™, Aquivion^®^) [39,42]. Traditional LSC membranes (e.g., Nafion^®^) offer high proton conductivity and excellent stability, making them the most widely used commercial PEMs. However, they are prone to dehydration at high temperatures (>100 °C), which leads to a significant decline in performance, and they also have poor dimensional stability [43,44]. In contrast, short-side-chain PFSA membranes (such as Aquivion^®^ and Hyflon^®^) demonstrate better applicability under medium-temperature operating conditions (approximately 120 °C) due to their higher glass transition temperature. In PEMWE applications, the advantages of Aquivion^®^ membranes are particularly prominent: their low equivalent weight, high crystallinity, and high glass transition temperature work together to achieve higher proton conductivity. This makes Aquivion^®^ comprehensively superior to Nafion^®^ in terms of performance, and its advantages are even more significant, especially when operating at high current densities [45,46,47]. Meanwhile, Aquivion^®^ features shorter side chains and a higher density of hydrophilic groups (-SO_3_H). This enables it to easily form continuous proton-conducting pathways. Additionally, it possesses high crystallinity (resulting in excellent thermal stability). Consequently, Aquivion^®^ outperforms Nafion^®^ in terms of water absorption rate and proton conductivity [48,49]. Molecular dynamics simulations have been used to compare the nanostructure and proton transport of Nafion and Hyflon [50]. While side-chain length has a minor effect on local structure, it significantly impacts phase separation and water channel topology, with Hyflon showing less phase separation. A key finding is that water and hydronium ion velocities increase from the polymer interface toward the channel center. Due to its shorter side chains, Hyflon has more mobile H_3_O+ ions in the channel center, resulting in higher diffusion coefficients for both water (25.2 × 10^−6^ cm^2^/s) and hydronium ions (6.5 × 10^−6^ cm^2^/s) compared to Nafion (21.3 × 10^−6^ cm^2^/s and 6.1 × 10^−6^ cm^2^/s), explaining its superior proton conductivity. Its further development and industrialization can be promoted through approaches such as composite modification, process optimization, and structural modeling.

The EW of PFSA membranes is a key parameter determining their comprehensive performance. By influencing the crystallinity of the membrane and the density of sulfonated side chains, it further affects proton conductivity, mechanical stability, chemical stability, and gas permeability. PFSA membranes with high EW exhibit high crystallinity, which endows the membranes with excellent mechanical strength and dimensional stability. However, high crystallinity restricts the connectivity of hydrophilic regions, hindering proton conduction and thereby reducing proton conductivity. Meanwhile, the closely arranged polymer chains also block the diffusion paths of gases such as hydrogen, leading to a decrease in the hydrogen permeability coefficient when the EW is excessively high (>909). In contrast, PFSA membranes with low EW show opposite characteristics. Owing to the higher density of sulfonated side chains, low-EW membranes can absorb more water and form more well-developed ion channels, thus significantly improving proton conductivity and hydrophilicity. Nevertheless, the higher density of sulfonated side chains increases the sites for free radical attack, making the side chains more prone to cleavage and thereby impairing the chemical stability of the membrane. Additionally, excessive water absorption and swelling also damage its mechanical and dimensional stability [31,51,52,53].

Despite the aforementioned challenges of low-EW membranes, their performance bottlenecks are being effectively addressed through structural innovation. For instance, PFSA membranes with a low-EW SSC structure leverage the excellent water retention capacity brought by high side-chain density to successfully balance high proton conductivity and high durability, making them a crucial direction for the optimization of PEM materials [54]. Furthermore, low-EW materials themselves have a higher sulfonic acid group density, which can shorten proton conduction paths and significantly reduce electrochemical polarization losses. Moreover, through a composite-reinforced structure, they can reduce membrane thickness while compensating for the insufficient mechanical strength of thin membranes, thereby meeting the mechanical requirements during the operation of PEMWE [55].

In summary, EW’s impact on PFSA membrane performance presents a “trade-off” relationship. In the low-EW range, a moderate increase in EW can improve the hydrogen permeability coefficient; however, when the EW is excessively high, the sharp increase in crystallinity becomes the dominant factor, which instead inhibits gas permeation. Therefore, in the design of PFSA membranes, it is essential to seek the optimal balance among EW, crystallinity, water content, and microstructure based on specific application requirements to achieve performance optimization.

#### 3.1.2. Partially Fluorinated Polymers

PFSA PEMs possess advantages such as high chemical/thermal stability, electrical conductivity, and mechanical strength. However, their high cost and environmental impact limit large-scale applications. Partially fluorinated PEMs are a type of membrane material improved based on PFSA PEMs. Specifically, only some carbon atoms in the polymer molecular chain are substituted by fluorine atoms. By reducing the fluorine content in the membrane material, their environmental impact and cost are lowered [56,57,58]. These membranes exhibit excellent proton conductivity as well as physicochemical stability; they can selectively allow proton permeation while blocking the passage of other ions, electrons, and gases, making them applicable in the field of PEMWE [59]. Currently, research on partially fluorinated PEM mainly focuses on balancing the membrane’s chemical stability, proton conductivity, gas barrier properties, and mechanical strength through the design of different molecular structures.

**Figure 3:**
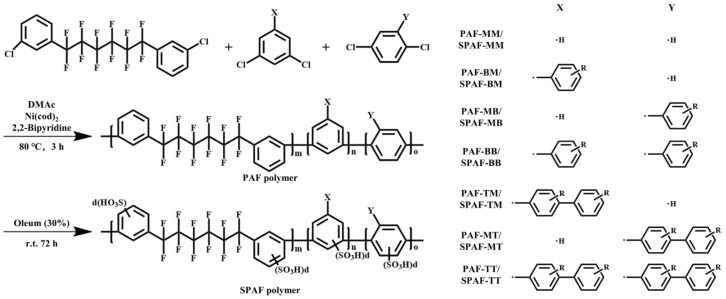
Synthesis and chemical structure of SPAF polymers. R = −H for PAF and R = −(SO_3_H)d for SPAF.

Six types of sulfonated poly(aryl perfluoroalkyl) (SPAF) membranes were successfully designed and synthesized by introducing pendant multisulfonated benzene groups into the SPAF polymer, whose structures are shown in Figure 3 [60]. Compared to the SPAF-MM membrane, the novel SPAF membranes exhibit a higher IEC of 2.07–2.15 meq/g. Compared to Nafion^®^ membranes, SPAF membranes exhibit superior chemical stability. For instance, during a 1000 h OCV hold test (80 °C, 30% RH), the SPAF-BM membrane showed a negligible decay of 40 μV h^−1^, with its molecular structure and weight remaining unchanged post-test, whereas Nafion^®^ membranes have low durability under OCV conditions. They also have lower gas permeability; the SPAF-BM membrane showed a significantly lower hydrogen crossover current density than Nafion^®^ at 80 °C.

**Figure 4:**
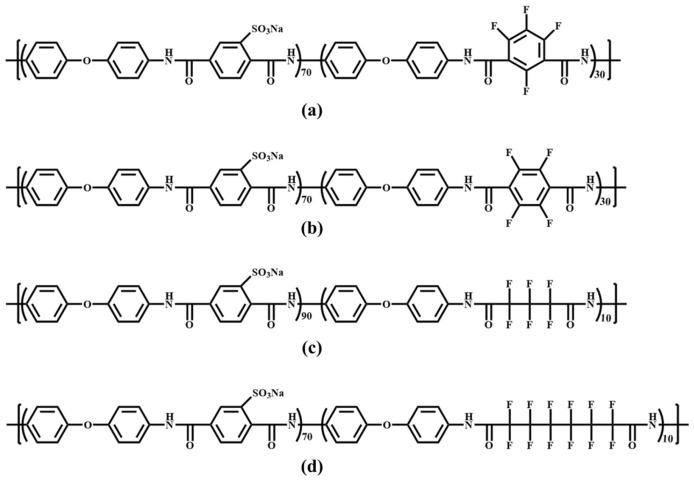
Structure of fluorinated sulfonated polyether amides: (**a**) ODA-STA-TFIPA-70, (**b**) ODA-STA-TFTPA-70, (**c**) ODA-STA-HFGA-90, (**d**) ODA-STA-PFSEA-90.

Partially fluorinated sulfonated polyetheramide (SPA) was synthesized via polycondensation, with a sulfonation degree of 80–90% and an IEC of 1.7–2.2 meq/g, whose structures are shown in Figure 4 [61]. Tough and transparent membranes were prepared using the dimethyl sulfoxide (DMSO) solution casting method. The results showed that fluorinated segments could reduce the membrane’s water absorption rate and improve its thermal stability. However, the low reactivity of fluorinated dicarboxylic acid led to a relatively low molecular weight of fluorinated SPA, which in turn resulted in lower hydrolytic stability compared to its hydrogenated counterpart. Under 80 °C and 100% RH, 90% sulfonated SPA membranes showed proton conductivities above 100 mS/cm.

**Figure 5:**
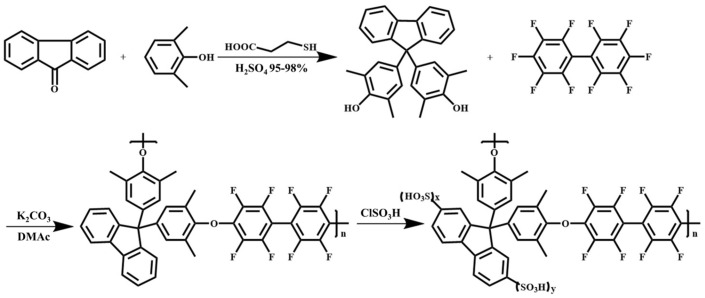
Synthesis of partially fluorinated sulfonated polyfluorene ether ketone.

A partially fluorinated sulfonated polyfluorene ether ketone (PFEK) was successfully synthesized, as shown in Figure 5, where sulfonation occurred exclusively at the (2,7)-positions of the fluorene groups [62]. This structure forms continuous proton transport channels, leading to higher proton conductivity; the fluorinated groups, with strong hydrophobicity and oxidation resistance, can enhance the membrane’s hydrophobicity, oxidation resistance, and hydrolytic stability. In addition, the main-chain structure of PFEK membranes is more stable than that of Nafion^®^ membranes, allowing them to resist the effects of high-temperature and oxidative environments. It revealed that the highly sulfonated membranes (with degrees of sulfonation of 1.75 and 2.00) exhibited higher proton conductivity (40 mS/cm and 57 mS/cm) than Nafion^®^ 117 (39 mS/cm), while the low-sulfonated membranes showed lower conductivity. The oxidative stability of the partially fluorinated membranes was superior to that of non-fluorinated membranes (although it decreased with increasing degree of sulfonation).

**Figure 6:**
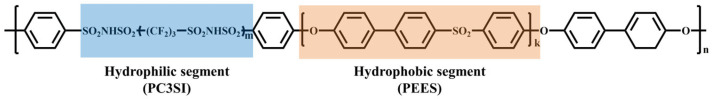
Chemical structure of perfluorocarbon–hydrocarbon hybrid block copolymer electrolyte.

A new type of fluorocarbon–hydrocarbon hybrid block copolymer electrolyte with polyperfluoropropyl sulfonylimide (PC3SI) as the hydrophilic segment and polyether ether sulfone (PEES) as the hydrophobic segment was synthesized, whose structure is shown in Figure 6 [63]. The hydrophilic segment (PC3SI) of the hybrid block copolymer membrane has an extremely low EW of 293 g/mol, which is conducive to forming more proton-conducting channels and thus improving proton conductivity; in addition, the sulfonylimide groups of PC3SI possess superacidity, which also contributes to enhancing proton conductivity. Its proton diffusion coefficient is approximately 1.5 times that of Nafion^®^ 112.

### 3.2. Non-Fluorine Polymers

#### 3.2.1. Heteroatom-Containing Polymers

To address the issues of high cost, fluorine pollution risk, high hydrogen permeability, and lack of stability at high temperatures associated with traditional PFSA membranes in PEMWE, the development of low-cost, high-performance non-fluorinated alternative materials has become a research focus [64,65,66]. Compared with perfluorinated ionomer membranes, some non-fluorinated hydrocarbon polymer membranes have lower production costs, more stable performance at high temperatures, greater environmental friendliness, and easier processability, but their chemical stability is usually reduced [67,68,69,70,71]. This type of material is typically aromatic polymers, mainly including sulfonated polyaryletherketone (SPAEK), sulfonated polyetheretherketone (SPEEK), sulfonated polysulfone (SPSF), sulfonated polyimide (SPI), sulfonated polyphenylene (SPP), and others [72]. The rigid backbone structure of aromatic polymers is the foundation for their excellent mechanical properties and thermal stability. The introduction of “-SO_3_H” via sulfonation reaction endows the material with the necessary proton conductivity. Additionally, aromatic polymers offer significant cost advantages due to the easy availability of raw materials and simple synthesis routes. According to the difference in elements composing the main chain, non-fluorinated PEMs can be divided into heteroatom-containing main-chain polymers and all-carbon main-chain polymers.

Non-fluorinated PEMs with heteroatom-containing backbones are one of the important research directions in the field of PEMs. Compared with PFSA-based PEMs, non-fluorinated PEMs with heteroatoms usually have cost advantages, as their raw materials are easily accessible and their processing technologies are relatively simple. However, the presence of heteroatoms, such as oxygen and sulfur, in their backbones creates electron-rich sites, which are vulnerable to free radical attacks in acidic operating environments, resulting in poor chemical stability [73,74].

**Figure 7:**
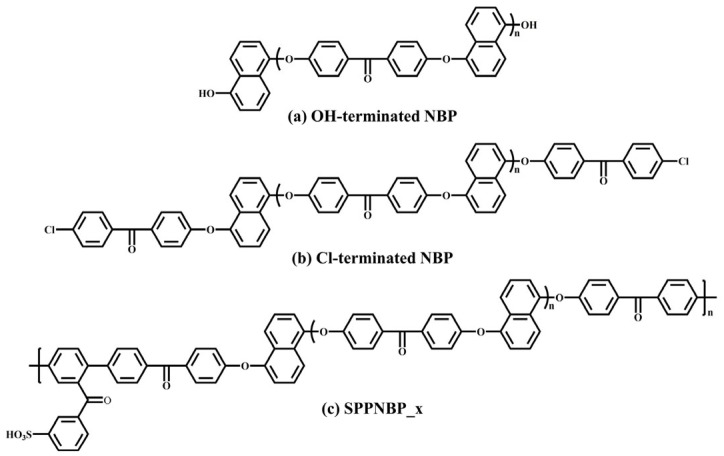
Chemical structures of (**a**) OH-terminated NBP, (**b**) Cl-terminated NBP, and (**c**) SPPNBP series.

Multi-block copolymer membranes (SPPNBP series) composed of SPP and naphthyl-containing polyaryletherketone (NBP) were synthesized via a nickel(0)-catalyzed coupling reaction, and their structures are shown in Figure 7 [75]. The introduction of naphthalene units enhances π-π interactions and increases the packing density of the hydrophobic regions, thereby reducing hydrogen permeability, suppressing excessive swelling, and maintaining mechanical stability. The multi-block structure promotes hydrophilic–hydrophobic microphase separation, forming continuous hydrophilic channels, which is beneficial for proton conduction. SPPNBP_5 showed proton conductivity comparable to Nafion^®^ 212 (200 vs. 196 mS/cm at 80 °C, 100% RH) and a hydrogen permeability over three times lower (<32 vs. 115.0 bar), and the proton/hydrogen selectivity of SPPNBP_3, 5, and 7 was approximately 3.6 times that of Nafion^®^ 212. After treatment with Fenton’s reagent, the residual weight (89.1–98.7%) was slightly lower than that of Nafion^®^ 212 (99.4%), but far superior to that of non-fluorinated membranes. In a PEMWE single cell, the current density of SPPNBP_5 at 1.9 V and 80 °C (5.5 A/cm^2^) was higher than that of Nafion^®^ 212 (4.75 A/cm^2^).

**Figure 8:**
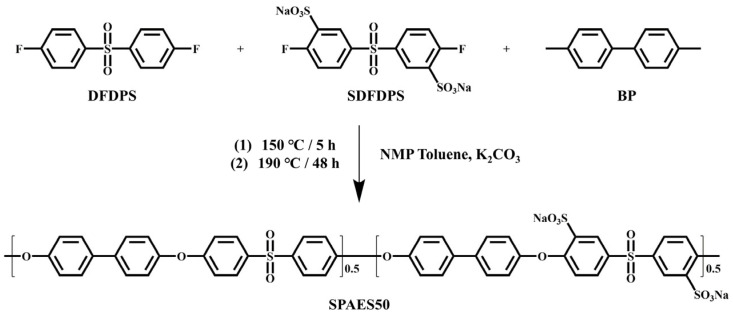
Synthesis of SPAES50 (DS = 50 mol%).

The sulfonated poly(arylene ether sulfone) with a 50 mol% sulfonation degree (SPAES50) was synthesized, as shown in Figure 8 [76]. The chemical structure of the SPAES50 membrane differs from that of the Nafion^®^ membrane: its sulfonic acid groups are more uniformly distributed, providing more proton conduction channels. Compared to Nafion membranes, the SPAES50 membrane with a degree of sulfonation of 50 mol% exhibited much higher proton conductivity (330.1 mS/cm at 90 °C) than Nafion 211. The hydrogen permeability of the 20 μm thick SPAES50 membrane was comparable to that of the 125 μm Nafion 115. However, SPAES50 showed a higher swelling ratio than Nafion 211, although it is lower in cost and free from fluorine pollution risks.

Among non-fluorinated PEMs with heteroatom-containing backbones, polybenzimidazole (PBI) is relatively unique. As a PEM, PBI exhibits outstanding performance under harsh conditions such as high temperatures (100–200 °C). Unlike PFSA membranes that rely on hydrated proton (H_3_O^+^) conduction and are different from traditional sulfonic acid membranes, PBI membranes require doping with proton carriers (e.g., phosphoric acid and sulfuric acid) to achieve proton conduction [77]. Meanwhile, PBI molecules possess amphoteric properties—they can be both protonated and deprotonated, which enables PBI membranes to have excellent ionic conductivity in both acidic and alkaline environments. This is of great significance for acid–alkaline amphoteric water electrolysis (AAA-WE).

To gain deep insights into the interaction between phosphoric acid (PA) and polybenzimidazoles (PBIs, including PBI, ABPBI, and PBDI) as well as the proton transfer (PT) mechanism, multi-scale simulation studies (quantum mechanics [QM], classical molecular dynamics [MD], and ADMP/ONIOM) have provided key molecular-level evidence [78]. The interaction between PA and benzimidazole is strong, and the PT energy barrier between the same molecules or a molecule and its corresponding ion is lower; the hydrogen bond strength follows the order of PA > water > polymer, with ABPBI and PBDI exhibiting superior hydrophilicity and PA affinity compared to PBI. Specifically, when the PA doping level reaches 2 molecules per imidazole ring, protonation occurs at the “=N-” atom on the imidazole ring; if 3 PA molecules are doped, the third PA can further promote protonation through bridging hydrogen bonds. In terms of diffusion performance, the molecular diffusion coefficient follows the order of water > PA > polymer. High temperature, high PA doping amount, and high water content all contribute to increasing the diffusion coefficient, and ADMP simulations verify that the proton diffusion coefficient of PA clusters is in good agreement with experimental values. Additionally, PA doping leads to an increase in membrane density and a decrease in chain spacing. Among these polymers, ABPBI demonstrates the best comprehensive performance due to its balanced hydrogen bond network and diffusion properties, providing a theoretical basis for the molecular design of high-temperature proton exchange membranes.

H_2_SO_4_-doped m-PBI membranes and OPBI membranes were prepared for AAA-WE [79]. These membranes exhibit good chemical stability, a low swelling ratio, moderate water absorption capacity in high-concentration acidic/alkaline solutions, and the ability to maintain favorable mechanical stability. Among them, m-PBI membranes perform better than Nafion^®^ 115 membranes in AAA-WE for hydrogen production. However, the phosphoric acid or sulfuric acid doped in PBI undergoes irreversible chemical degradation in the strong oxidizing environment of the water electrolysis anode, leading to membrane structure damage and functional failure. Additionally, the doped acid is easily leached by water, which further reduces proton conductivity and prevents PBI membranes from meeting the long-term stable operation requirements of water electrolysis for hydrogen production [80].

PBI-Gel membranes with a porous layered structure were prepared via the polyphosphoric acid (PPA) sol–gel method to improve the membrane’s porosity and adsorption capacity [81]. Subsequently, through a “2D polarization/1D shrinkage” process (clamping the in-plane dimensions and allowing only cross-membrane shrinkage for 24 h), the PBI-Gel membranes were converted into PBI-aNS membranes composed of hundreds of dense nanosheets with a thickness of 28 nm. This modification enhances the membrane’s ion transport capacity, anti-cross-contamination ability, and mechanical properties.

Thermally cured PBI membranes were selected to improve their oxidation and corrosion resistance [82]. By controlling the operating temperature, the occurrence of side reactions caused by excessively high temperatures was avoided. When phosphoric acid-doped PBI membranes operate below 130 °C, the formation of sulfur can be effectively controlled, and the membranes exhibit good chemical stability and electrochemical performance.

#### 3.2.2. All-Carbon Backbone Polymers

Currently, commonly used PFSA polymer membranes have drawbacks such as low glass transition temperature and complex synthesis processes. Phosphoric acid (PA)-doped PBI membranes, on the other hand, face issues including poor solubility and the migration and loss of PA. Alternative materials like SPEEK exhibit insufficient chemical stability due to the presence of heteroatoms in their main chains, which makes them vulnerable to free radical attacks. Aromatic polymers with all-carbon backbones (e.g., polyphenylenes, phenylated polyphenylenes, and poly(arylene alkane)s) have emerged as highly promising alternative materials for PEMs, thanks to their excellent chemical stability, thermal stability, mechanical properties, and high proton conductivity after the introduction of acidic groups [83,84]. However, non-fluorinated membranes also face multiple challenges: it is difficult to balance proton conductivity and mechanical stability; they are prone to dehydration at high temperatures, leading to a sharp drop in conductivity; and after sulfonation, they are vulnerable to hydroxyl radical attacks, resulting in a relatively short membrane lifespan [85]. Therefore, designing new structures to address the above issues has become a research focus in this field.

**Figure 9:**
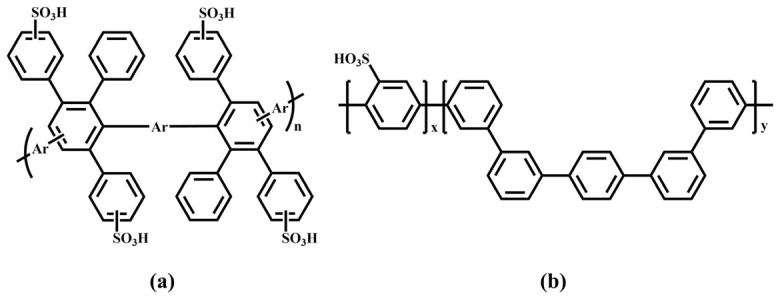
Chemical structures of Pemion^®^ (**a**) and SPP-QP (**b**).

Pemion^®^ membrane is a breakthrough proton exchange membrane and polymer product developed by Ionomer Innovations (Canada), and it is a representative of all-carbon backbone polymer membranes, as shown in Figure 9a.

The Pemion^®^ membrane’s higher IEC (2.56–2.85 mmol g^−1^) and water uptake, relative to Nafion^®^, afford it much greater proton conductivity. Pemion^®^ has a rigid aromatic ring structure, which restricts the movement of polymer segments, allowing it to maintain high mechanical strength even at high temperatures. Although Pemion^®^ has a relatively high water absorption rate, its rigid backbone limits the plasticizing effect of water molecules on the material. Additionally, the rigid aromatic backbone of Pemion^®^ reduces gas permeation, thereby improving safety [86,87,88,89].

Pemion^®^ membranes were compared with traditional Nafion^®^ membranes and non-reinforced sPPB-50 membranes [90]. The results showed that Pemion^®^ membranes possess excellent proton conductivity, low hydrogen permeability, and good durability, making them a sustainable alternative to traditional Nafion^®^ membranes. It was found that the Pemion^®^ membrane, with its rigid polyphenylene backbone, exhibits excellent high-temperature resistance [91]. Its Young’s modulus and strain hardening are hardly affected by temperature, while the mechanical properties of traditional reinforced PFSA (r-PFSA) membranes significantly decline above 90 °C. In addition, DSC and TGA analyses confirm that the Pemion^®^ membrane has no significant phase transition or decomposition below 300 °C, indicating excellent thermal stability.

## 4. PEM Structure Design

### 4.1. Homogeneous PEM

PEMs can be classified into homogeneous membranes and composite membranes based on their structure. Homogeneous membranes are composed of a single material (e.g., Nafion^®^, Aquivion^®^), featuring mature technology and high conductivity. However, they suffer from high costs and, when used in water electrolysis for hydrogen production, exhibit the issue of high hydrogen permeability. The properties of pure homogeneous membranes can be improved through heat treatment, surface treatment, and other methods [92]. However, to fundamentally overcome these limitations, membrane materials with more excellent comprehensive performance can be prepared by compounding with different materials. There are two main specific composite methods. One method is compounding with organic polymers, for example, by combining a proton-conducting layer (such as PFSA materials) with a support layer (such as polytetrafluoroethylene, PTFE) or cross-linking to form an interpenetrating network [93]. This approach improves the mechanical stability of the membrane through the support layer while retaining the ion transport performance of the proton-conducting layer. The other method is compounding by introducing inorganic nanofillers or organic modifiers, where inorganic nanofillers such as graphene oxide (GO), metal–organic frameworks (MOFs), and metal oxides can be added. Through the synergistic effect between the nanofillers and the matrix material, key properties of the membrane, such as proton conductivity and thermal stability, are optimized [85].

### 4.2. Composite PEM

#### 4.2.1. Polymer Blends

The preparation of PEMs via organic polymer composite technology enables the synergistic enhancement of multi-dimensional core properties of membrane materials. Using organic polymers as the reinforcing phase to construct a composite system with a PFSA matrix can effectively make up for the mechanical performance shortcomings of PFSA membranes, addressing the issue of easy deformation or damage during long-term service. Meanwhile, some organic polymer components can endow the composite membrane with excellent environmental resistance, allowing it to maintain structural stability even under harsh operating conditions, such as high temperatures and acidic or alkaline environments. Additionally, the composite of specific organic polymers with the matrix can regulate the membrane’s microstructure (e.g., constructing a dense molecular chain network), which greatly inhibits the cross-permeation of gases such as hydrogen. This optimizes the membrane’s gas barrier properties and provides a guarantee for the efficient and stable operation of application scenarios such as fuel cells.

An enhanced composite membrane (SPP-QP-PE) composed of self-made SPP ionomer (SPP-QP) and flexible porous polyethylene (PE) substrate was prepared via the doctor blading method [94]. The SPP-QP completely filled the pores of PE, significantly enhancing its overall performance through synergistic effects, interfacial interactions, and pore filling. The proton conductivity was enhanced because SPP-QP with high IEC provided more proton transport channels. The gas impermeability was comparable to that of the bare SPP-QP membrane and much lower than that of the PFSA Nafion^®^ membrane, effectively preventing hydrogen–oxygen cross-permeation and improving fuel cell safety. Overall, the membrane achieved an excellent balance in proton conduction and gas barrier properties.

**Figure 10:**
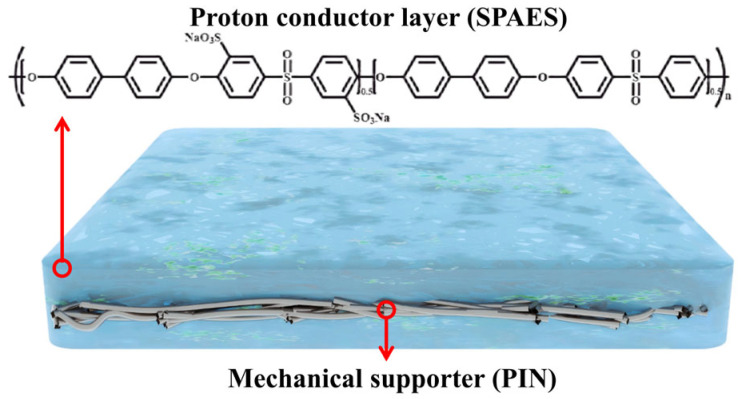
Structure of sulfonated poly(arylene ether sulfone)/polyimide nanofiber (SPAES/PIN) composite PEM.

An SPAES/PIN composite PEM was developed, whose structure is shown in Figure 10 [95]. SPAES, acting as the proton-conducting polymer, provides high proton conductivity and selectivity, while PIN, serving as the mechanical reinforcement layer, effectively enhances the membrane’s mechanical strength (tensile strength exceeding 40 MPa) and dimensional stability (area swelling ratio of only 7.5%). The composite membrane exhibits excellent proton selectivity (proton transport number of 0.96), with a permeability to competing cations 3–10 times lower than that of Nafion^®^ 211, while significantly reducing the permeability of gases and substrates. In microbial electrolysis cell (MEC) applications, this composite membrane increases hydrogen production by 32.4% with a hydrogen purity of 90.3% and alleviates the pH gradient problem caused by proton accumulation at the anode (ΔpH = 1.5). Additionally, the internal resistance of the composite membrane is 49.5 Ω lower than that of Nafion^®^ 211, further improving system efficiency.

A modified PEEK mesh, which had undergone oxygen treatment and molybdenum disulfide (MoS_2_) sputtering treatment, was embedded into the SPEEK matrix [96]. By introducing the MoS_2_-sputtered modified MPEEK (modified polyether ether ketone) mesh into the SPEEK matrix, the composite structure significantly enhanced the overall performance of the membrane: The mechanical strength increased from 34.3 MPa (of pure SPEEK) to 48.9 MPa. The dimensional stability was greatly improved, with the swelling rate at 90 °C decreasing from 45.5% to 2.86%. Although the proton conductivity decreased slightly, the SPEEK-MPEEK membrane still maintained a high level (42.5 mS/cm), which benefited from the optimization of proton transport paths by MoS_2_. In addition, the free radical scavenging effect of MoS_2_ significantly enhanced the oxidative stability of the membrane, with the residual weight reaching 86.9%.

**Figure 11:**
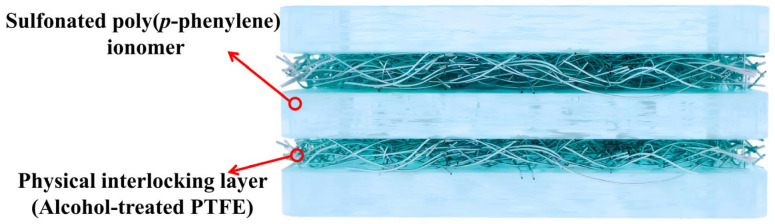
Structure of the five-layered sulfonated poly(p-phenylene) (SPP) ionomer/PTFE.

An SPP-based ionomer/PTFE five-layer composite membrane for PEMFCs and PEMWEs was developed, with its structure illustrated in Figure 11 [97]. Compared to the Nafion 212 membrane, the SPP/PTFE five-layer composite membrane exhibits numerous performance advantages. Its PTFE matrix effectively suppresses the excessive swelling of the SPP ion exchanger with a high IEC in water, resulting in a significantly lower in-plane swelling ratio at 80 °C (10.7%) than that of Nafion 212 (14.6%), thereby achieving superior dimensional stability. Concurrently, the excellent mechanical strength of PTFE imparts the composite membrane with greater mechanical strength and durability, enabling it to withstand 4800 dry–wet cycles in a PEMFC without failure. Furthermore, the gas barrier properties of PTFE reduce the hydrogen permeability to approximately 1/4.9 of that of Nafion 212, decreasing fuel loss and safety risks. In terms of electrochemical performance, the composite membrane’s thinner structure lowers the cell’s internal resistance, and the suppression of swelling optimizes the interfacial contact between the membrane and the catalyst layer, leading to higher proton conductivity at 80 °C than Nafion 212. This, in turn, drives a 16% increase in current density in PEMWE. Although the voltage decay rate during the 100 h PEMWE test is higher than that of Nafion 212, it remains superior to that of the pure SPP-2.0 membrane.

#### 4.2.2. Nanofillers

In PEMWE, the preparation of PEMs through compounding with inorganic nanofillers can specifically address the performance limitations of pure organic membranes under harsh electrolytic environments. Common materials used for modification include metal oxides, carbon nanotubes (CNTs), GOs, MOFs, etc. [98,99]. Compounding with inorganic nanofillers enhances PEM performance by (1) enhancing proton conductivity: inorganic nanofillers construct supplementary proton channels via their own hydroxyl groups and acid sites or improve water retention capacity through porous structures; (2) improving oxidation resistance and chemical stability: inorganic nanofillers can trap free radicals or form structural barriers to reduce the attack of active free radicals; (3) optimizing mechanical and dimensional stability: rigid fillers enhance tensile strength and creep resistance, while lamellar fillers inhibit excessive water absorption; (4) reducing gas permeability: lamellar fillers form tortuous permeation paths to block gas diffusion; and (5) adapting to the operating requirements of a wide temperature range: nanofillers with high thermal stability expand the working temperature range, alleviate mass transfer limitations, and prevent thermal degradation. Therefore, the comprehensive performance and service life of the membrane are fully improved [85,100,101].

An SPVDF/ZrP composite membrane was prepared using PVDF-co-HFP as a matrix, which was subjected to an alkali treatment for defluorination and sulfonation to render it proton-conductive, and was then incorporated with inorganic zirconium phosphate (ZrP) nanoparticles [102]. By incorporating the hydrophilic inorganic material ZrP, the membrane effectively increases proton exchange sites and enhances hydrophilicity, thereby significantly improving proton conductivity. Its conductivity (0.086 S/cm), although slightly lower than that of Nafion^®^117, is very close. In practical hydrogen production, the voltage and efficiency of the two are comparable, and the SPVDF_5_/ZrP_1_ membrane can increase the hydrogen production rate at higher current densities. More importantly, due to the hydrophobicity of the PVDF matrix, the membrane possesses superior mechanical strength with a higher Young’s modulus (299.36 MPa vs. 112.8 MPa). The introduction of ZrP also enhances the membrane’s stability, maintaining a stable voltage during 20 h of operation. The SPVDF/ZrP membrane is expected to be an alternative to Nafion^®^ for hydrogen production in ECMR.

**Figure 12:**
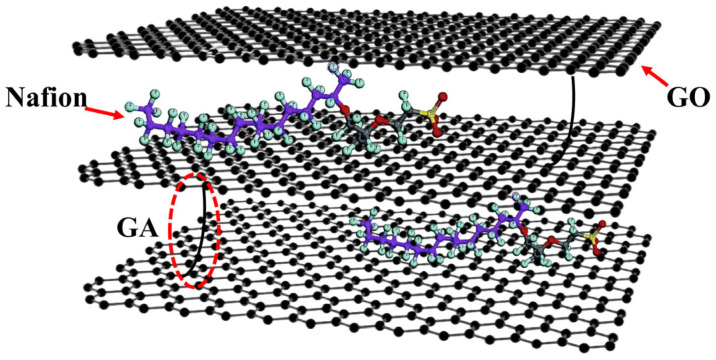
Composite structure of the GO/Nafion^®^-X membranes.

A GO/Nafion^®^ composite membrane was developed, as shown in Figure 12 [103]. The horizontal stacking of GO nanosheets forms long-range ordered interlayer nanochannels, and the embedded Nafion^®^ ionomer regulates the interlayer spacing, hydrogen bond network, and other microenvironments through sulfonic acid groups to promote proton transport. Meanwhile, glutaraldehyde (GA) cross-links the membrane to enhance its structural stability. The proton conductivity of the GO/Nafion^®^-75% membrane (314 mS cm^−1^ at 80 °C, 98% RH) exceeds that of Nafion^®^ 117. Additionally, it maintains good stability after soaking in water for 30 d and possesses excellent hydrogen barrier properties.

**Figure 13:**
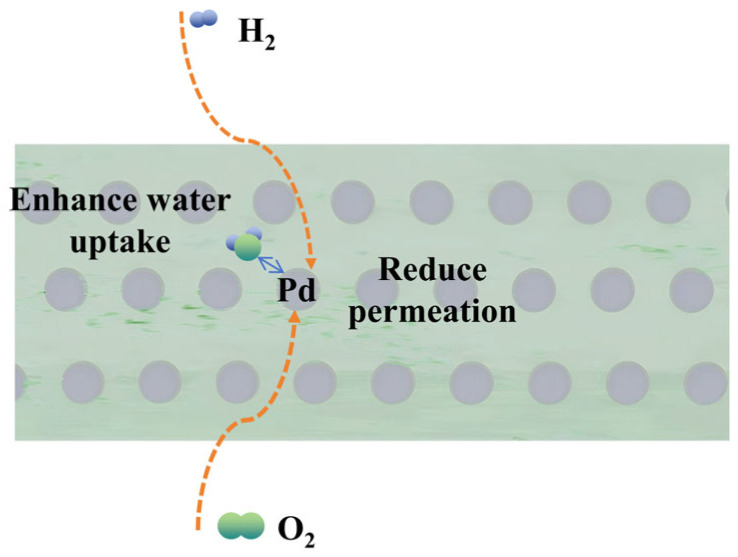
Schematic of the function of Pd-loaded membranes on hydrogen crossover.

Nafion^®^-Pd composite membrane (nano-Pd-loaded) was prepared by recasting, as shown in Figure 13 [104], aiming to reduce hydrogen permeation in PEMWE. The membrane exhibits slightly lower IEC than commercial Nafion^®^ 115 but markedly improved water uptake and hydration degree, increasing the proton conductivity by 24.8%. Additionally, it exhibits better oxidation stability and thermal stability, with a slight decrease in mechanical strength. Nafion^®^-Pd membrane shows drastically reduced hydrogen permeation (0.66% H_2_ in O_2_ at 0.1 A/cm^2^)—60.2% lower than Nafion^®^ 115 and superior to Pt-doped membranes. Stable electrolytic voltage and H_2_ concentration during long-term operation offer an effective solution to PEMWE hydrogen permeation safety concerns.

Highly dispersed platinum nanoparticles (Pt NPs) with an average diameter of approximately 3 nm were prepared [105], and they were precisely embedded in the hydrophilic domains of an ultra-thin double-layer PFSA membrane. Meanwhile, traditional Pt black-doped membranes (CM/Pt series) were prepared for comparison. This precise doping strategy achieved efficient hydrogen crossover suppression under an ultra-low Pt loading of 0.002 mg cm^−2^. Compared with the traditional Pt doping method, precise doping avoids Pt aggregation and improves Pt utilization efficiency, thereby enabling more effective hydrogen elimination. In a practical PEMWE device, the membrane loaded with 0.1% Pt maintained stable hydrogen crossover suppression performance after continuous operation for 500 h at current densities of 1.0 and 2.0 A·cm^−2^, demonstrating the excellent durability of the membrane.

**Figure 14:**
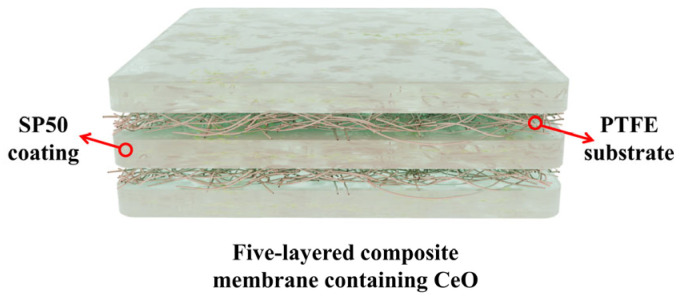
Composite structure of SP50/PTFE membranes.

A five-layer hydrocarbon ionomer/PTFE composite membrane (F-SP50-Ce) containing cerium oxide (CeO_2_) as a free radical scavenger was developed for application in PEMWE, as shown in Figure 14 [106]. CeO_2_ was uniformly dispersed via ball milling, and its core functions are remarkable. First, it scavenges free radicals such as •OH and •OOH through the Ce^3+^/Ce^4+^ redox cycle, thereby enhancing chemical stability. After a 100 h durability test, the membrane’s molecular weight only decreased by 5% (compared to a 50% decrease for pure SP50), and the degradation rate was as low as 50 μV/h (lower than that of Nafion^®^ 212). Second, it assists in enhancing physical properties: it works synergistically with PTFE to reduce the membrane’s volume change to 79.2% at 80 °C, and through physical barrier effects, the hydrogen permeability (25.3 barrer) is only 1/3.8 that of Nafion^®^ 212. This membrane achieves a PEMWE current density of 7.42 A/cm^2^ at 80 °C and 1.9 V (31% higher than that of Nafion^®^ 212), and its proton conductivity (0.142 S/cm at 80 °C) is still superior to that of Nafion^®^ 212, balancing both stability and electrochemical performance.

**Figure 15:**
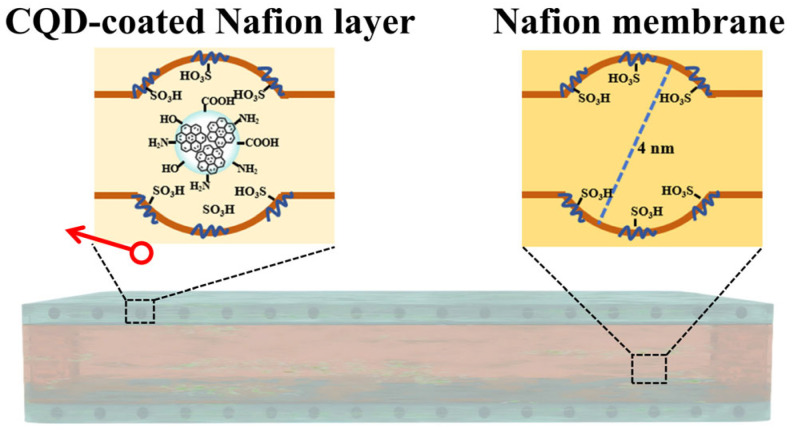
Composite structure of the N-doped CQD-coated Nafion^®^ membranes.

CQD-cNafion^®^ membranes were prepared by coating Nafion^®^-212 with hydrophilic nitrogen-doped carbon quantum dots (CQDs), as shown in Figure 15 [107]. The hydrophilic functional groups in CQDs enhance the membrane’s IEC and water absorption rate. Meanwhile, CQDs promote phase separation of the Nafion^®^ membrane, forming larger ion clusters, which significantly improves proton transport efficiency—this enables the membrane to achieve an ionic conductivity of 241.1 mS cm^−1^ at 80 °C. In addition, the incorporation of CQDs fills the pores of the membrane, effectively reducing hydrogen permeability by approximately 10%, and greatly enhances the membrane’s chemical stability by virtue of its free radical scavenging ability.

We summarize the advantages and disadvantages of different types of PEM mentioned in Chapters 3 and 4, as shown in Table 1.

## 5. Conclusions and Outlook

This study focused on PEM—the core component of PEMWE technology for hydrogen production—and emphasized the correlation between the microstructure and performance of membrane materials by analyzing the molecular structure design and modification strategies for the overall membrane structure to address the trade-off issues concerning the ionic conductivity, hydrogen permeability, and stability.

At the molecular structure design level of membrane materials, fluorinated and non-fluorinated PEMs exhibit distinct optimization pathways and application potential through synergistic effects of main chains, side chains, and sulfonate groups. Among fluorinated membranes, perfluorinated PEMs (e.g., Nafion^®^ and Aquivion^®^) leverage the phase-separated structure of PTFE backbones and sulfonic acid side chains, delivering high proton conductivity and chemical stability. Current research achieves performance breakthroughs by adjusting side-chain length (shortened side chains enhance applicability at medium temperatures) and equivalent weight (balancing proton conductivity, mechanical stability, and gas permeability). Partially fluorinated membranes (e.g., SPAF and SPA series) reduce costs and environmental impact by decreasing fluorine content. Relying on the stability of fluoroalkyl groups and the conductivity of sulfonated groups, they exhibit excellent durability and gas barrier properties, offering a cost-effective alternative to perfluorinated membranes. For non-fluorinated membranes, polymers with heteroatom-containing backbones (e.g., SPPNBP and SPAES) perform well in terms of proton conductivity, mechanical strength, and gas barrier properties, but their chemical stability still needs improvement. Polymers with all-carbon backbones (e.g., Pemion^®^) possess outstanding proton conductivity, mechanical strength, and high-temperature resistance, making them highly promising alternative materials.

Composite strategies effectively address the performance shortcomings of homogeneous membranes through multi-component integrations and synergistic effects. Composite with organic polymers: This approach leverages the synergy of “proton-conducting layer + support layer” (e.g., the sandwich structure of SPP-QP/PE, nanofiber reinforcement of SPAES/PIN, and five-layer composite of SPP/PTFE). While retaining high proton conductivity, it significantly enhances mechanical strength, dimensional stability, and gas barrier properties and reduces membrane internal resistance and hydrogen permeation risk. Composite with inorganic nanofillers: This targets specific performance defects for precise optimization. Nanofillers such as metal oxides (ZrO_2_ and CeO_2_), GO, and MOFs enable comprehensive improvements in proton conductivity, oxidation resistance stability, mechanical properties, and wide temperature adaptability through multiple mechanisms: constructing supplementary proton channels (via hydroxyl sites of ZrP), scavenging free radicals (via the redox cycle of CeO_2_), optimizing microstructures (via interlayer nanochannels of GO), and inhibiting hydrogen permeation (via the catalytic effect of Pd and Pt nanoparticles). Examples include the high conductivity of GO/Nafion^®^ composite membranes and the low hydrogen permeation of Nafion^®^-Pd membranes, which provide effective solutions for membrane performance optimization under harsh electrolytic environments.

In conclusion, the performance optimization of PEMs must focus on the establishment of “structure–property” correlation. At the molecular design level, it is necessary to balance proton conductivity, chemical stability, and mechanical strength; at the overall structural level, composite strategies should be adopted to overcome the limitations of single materials. Through in-depth learning of the correlation between the microstructure and performance of membrane materials and developing new materials and composite membrane structures, the performance of PEMs can be effectively improved. This will promote the large-scale application of hydrogen energy and contribute to solving global energy and environmental issues.

The substitution of perfluorinated compounds with all-aromatic polymers represents a significant trend in the development of proton exchange membrane (PEM) materials. Future research should focus on three core areas: developing novel all-aromatic polymers through molecular structure design to achieve materials with higher proton conductivity, longer lifespan, and enhanced stability; optimizing composite modification strategies by exploring more effective methods, such as the precise doping of nanofillers and organic/inorganic hybridization, to further improve the comprehensive performance of all-aromatic polymers; and establishing a performance evaluation system with scientific and comprehensive standards to fully assess all performance metrics of all-aromatic polymer PEMs, thereby providing theoretical guidance for their practical application. It is believed that with continuous technological advancements, all-aromatic polymer PEMs will see wider applications and make greater contributions to the development of clean energy.

## Figures and Tables

**Table 1:** Advantages and disadvantages of different types of polymers for proton exchange membranes.

Classification	Materials	Advantages	Disadvantages
PFSA	Nafion^®^	1. Stability: PTFE resists strong acids/high oxidation potentials. 2. Proton conductivity: >0.1 S/cm at 80 °C (continuous channels from phase separation). 3. Commercial maturity: Stable process, compatible with existing PEMWE.	1. High cost: Perfluorinated monomers are hard to synthesize; Nafion^®^-type is expensive. 2. Limited high-temperature performance: Dehydration >100 °C→sharp conductivity drop. 3. High H_2_ permeability: Nafion^®^ 212 (115 barrer). 4. Dimensional stability: High swelling at high humidity (affects membrane–electrode contact).
Partially fluorinated polymers	SPAF series, SPA series, PFEK	1. Cost–environmental balance: Reduced F content, lower cost vs. perfluorinated membranes, less F pollution. 2. Excellent gas barrier: SPAF-BM < Nafion^®^ in H_2_ permeation current density. 3. High structural tunability: Optimize conductivity/stability via sulfonation degree/fluoro chain length.	1. Inferior chemical stability: Lower F content→weaker radical resistance vs. perfluorinated membranes. 2. Complex synthesis: Low reactivity of some fluorinated monomers (e.g., SPA series fluorinated dicarboxylic acids)→low molecular weight. 3. Limited large-scale use: Lab-scale studies dominate; no industrial validation data.
Heteroatom-containing polymers	SPPNBP series, SPAES series, PBI	1. Ultra-low cost: 1/5–1/10 of perfluorinated membranes. 2. High conductivity: SPAES50 (330.1 mS/cm at 90 °C) > Nafion^®^. 3. High selectivity: SPPNBP-5 (3.6× vs. Nafion^®^ 212)→less gas crossover. 4. PBI high-T resistance: 100–200 °C, suitable for high-T PEMWE.	1. Poor chemical stability: Backbone with O/S heteroatoms→susceptible to ·OH/OOH attack. 2. High swelling: Excessive water uptake from a high sulfonation degree impairs structural integrity. 3. PBI needs acid doping: Relies on phosphoric/sulfuric acid for conductivity; acid leakage causes performance decay and electrode corrosion.
All-carbon backbone polymers	Pemion^®^	1. Stability breakthrough: Fully carbon aromatic backbone→better radical resistance vs. heteroatom-containing non-fluorinated membranes. 2. High-T mechanical excellence: Rigid framework inhibits thermal deformation > traditional reinforced PFSA membranes. 3. High conductivity + low permeability: IEC 2.56–2.85 mmol/g; H_2_ permeability < Nafion^®^. 4. Eco-friendly and low cost: No F pollution, cheap raw materials, lower cost than perfluorinated membranes.	1. High water absorption risk: High IEC causes excessive water uptake. 2. Scalability technology to be improved: Current preparation is mainly lab-scale; lacks stability data for large-scale production. 3. Catalyst compatibility needs optimization: Low surface polarity of some all-carbon membranes may affect the membrane–catalyst layer interface contact.
Composite PEM	SPP-QP-PE, SPAES/PIN, GO/Nafion^®^, F-SP50-Ce	1. Synergistic optimization: Organic blending (SPP-QP/PE): High conductivity + PE matrix→mechanical strength >30% higher. Inorganic filling (GO/Nafion^®^): GO→314 mS/cm; CeO_2_→free radical scavenging, longer lifespan. 2. Targeted mitigation: Nafion^®^-Pd→H_2_ permeation ↓60.2%; F-SP50-Ce→current density ↑31% (vs. Nafion^®^ 212). 3. Controllable cost: Low-cost substrates (PTFE/PE) or small fillers (GO/CeO_2_)→avoid high costs associated with perfluorinated membranes.	1. Poor interfacial compatibility: Organic-inorganic phase separation→defects (nanofiller aggregation→lower conductivity). 2. Complex preparation: Multilayer composites (SPP/PTFE five-layer) and precise doping (Pt nanoparticle embedding)→high-precision equipment required. 3. Unverified long-term stability: Weak interfacial adhesion (e.g., CQD-cNafion^®^)→interlayer delamination during long-term operation.

## Data Availability

No new data were created or analyzed in this study.
